# Understanding women’s preferences for long-acting reversible contraceptives in Gondar, Ethiopia: a discrete choice experiment

**DOI:** 10.1186/s13561-025-00683-y

**Published:** 2025-10-21

**Authors:** Ousman Ambaw, Amare Minyhun, Tsegaw Amare Baykeda, Haimanot Wubale Tewabe, Endalew Minwuye Andargie, Yihalem Abebe Belay, Dessie Tarko Ambaw, Lei Si

**Affiliations:** 1 Department of Health Systems and Policy, Institute of Public Health, University of Gondar, Gondar, Ethiopia; 2 School of Public Health, University of Queensland,, Brisbane, Australia; 3 Department of Public Health, School of Public Health, Asrat Woldeyes Health Science Campus, Debre Berhan University, Debre Berhan, Ethiopia; 4 Department of Public Health, College of Medicine and Health Sciences, Debre Markos University, Debre-Markos, Ethiopia; 5 UniSA Business, University of South Australia, Adelaide, Australia; 6 School of Health Sciences, Western Sydney University, Campbell town, NSW Australia; 7 Translational Health Research Institute, Western Sydney University, Penrith, NSW Australia

**Keywords:** Long-acting reversible contraceptives, Contraceptive preferences, Discrete choice experiment, Willingness to pay, Ethiopia

## Abstract

**Background:**

In Ethiopia, limited use of long-acting reversible contraceptives (LARCs) contributes to unintended pregnancies, unsafe abortions, and preventable maternal deaths. Despite their proven effectiveness, LARCs remain underutilized. Evidence on women’s preferences and willingness to pay (WTP) is scarce. This study examined women’s stated preferences, WTP, and trade-offs regarding LARC use in Gondar.

**Methods:**

An institution-based cross-sectional study was conducted among 344 contraceptive users, generating 8,256 observations. A discrete choice experiment (DCE) with 24 choice tasks, divided into two blocks, was employed. Each task presented two unlabeled alternatives defined by six key attributes, identified through literature review and expert consultation. Data were analyzed using mixed logit models to estimate preference strength and WTP based on model coefficients.

**Results:**

The analysis revealed that provider type significantly influenced women’s preferences. Women showed the highest WTP for LARCs provided by midwives [528 ETB (10.15 USD)], compared to services offered by doctors [285 ETB (5.48 USD)] and health officers [215 ETB (4.13 USD)]. Preferences were also shaped by side-effect profiles: methods associated with slight weight gain [155 ETB (2.98 USD)], high effectiveness [80 ETB (1.54 USD)], and absence of bleeding [74 ETB (1.43 USD)] were positively valued. Conversely, heavy menstrual bleeding led to the largest reduction in WTP [–688 ETB (–13.24 USD)], indicating a significant barrier to LARC uptake. Longer-acting methods also reduced WTP [–139 ETB (–2.68 USD)], possibly reflecting concerns about long-term commitment or side effects. Cost sensitivity was evident, as increases of 100 ETB (1.92 USD) or 500 ETB (9.92 USD) further reduced uptake likelihood.

**Conclusion:**

Women’s preferences for LARCs are influenced by provider type, side effects, and cost. Enhancing LARC services by prioritizing midwife-led delivery, addressing side effects such as heavy menstrual bleeding, and considering women’s WTP can increase uptake. These findings highlight the need for affordable, user-centered contraceptive services in Ethiopia.

**Supplementary Information:**

The online version contains supplementary material available at 10.1186/s13561-025-00683-y.

## Background

Globally, although maternal mortality declined by 44% over the past 25 years, this remains short of the Millennium Development Goal (MDG) target of 75%, and maternal deaths remain a significant public health concern [1]. Effective contraception plays a critical role in reducing unintended pregnancies and maternal mortality. Long-acting reversible contraceptive methods (LARCs), such as intrauterine devices (IUDs) and contraceptive implants, are among the most effective contraceptive options, with failure rates of less than 1% [2]. However, in Sub-Saharan Africa (SSA), where there is comparatively low LARC use, addressing unmet contraception needs, particularly among young women, remains a challenge [3–6]. While the World Health Organization (WHO) recommends LARCs for all women, multiple barriers—including higher upfront costs, fear of side effects, and preference for short-term methods—limit their utilization, especially in low-income countries like Ethiopia, where existing subsidies have not significantly increased LARC uptake [7–12].

Ethiopia, the second most populous country in SSA, has a fertility rate of 4.1 children per woman and a modern contraceptive prevalence rate of 29% [13]. However, LARC utilization remains low: national estimates indicate approximately 10–20% usage, compared to 35% for short-acting methods [13–15]. Unplanned pregnancies are a prevalent issue in Ethiopia, often linked to marital status and the number of sexual partners. This highlights the importance of reliable contraception methods to prevent unintended pregnancies [16]. Unplanned pregnancies remain widespread, contributing to maternal health risks and underscoring the need for more reliable contraceptive methods [17]. Despite their effectiveness, scientific evidence on Ethiopian women’s preferences for LARCs remains limited.

Existing research in Ethiopia has predominantly focused on identifying general barriers and facilitators of contraceptive use using descriptive surveys and qualitative methods [18–20]. While informative, these methods fail to quantify how women weigh trade-offs between attributes such as side effects, cost, and provider type, or their willingness to pay (WTP) for improved service features. Importantly, there is little evidence on attribute-level preferences or economic valuation of LARC characteristics, which limits policy-makers’ ability to design demand-driven interventions.

To address these gaps, this study applies a Discrete Choice Experiment (DCE), a stated preference method increasingly used in health economics to quantify individual preferences in contexts where actual behavior may not reveal true demand [21]. DCE presents hypothetical choice sets composed of alternatives varying across several attributes, requiring respondents to select their preferred option. This approach allows quantification of trade-offs, attribute importance, and WTP, offering policy-relevant insights for contraceptive service design and financing strategies [21, 22].

Furthermore, this study is conceptually grounded in random utility theory (RUT**)** and economic rational choice theory. According to RUT, individuals make decisions that maximize their perceived utility, which is determined by both observable attributes (e.g., cost, effectiveness, provider type) and unobserved factors [23]. Similarly, economic rational choice theory assumes that individuals are rational actors who make decisions by comparing the benefits and costs of available alternatives and selecting the option that offers the highest personal utility. In the context of contraceptive choices, this theory suggests that women evaluate each method based on its characteristics such as side effects, duration, cost, and provider and choose the one that best aligns with their preferences and circumstances [24]. Applied in this study, women are assumed to choose the contraceptive method that maximizes their perceived benefits relative to cost, with choices modeled to estimate preferences, trade-offs, and WTP [25]. DCE methods, underpinned by utility maximization theory, are thus particularly suitable for exploring contraceptive preferences in this context [26].

International evidence highlights variability in contraceptive preferences, shaped by factors such as side effects, provider recommendations, and service accessibility [27–31]. In Ethiopia, although previous studies have explored general barriers to the uptake of long-acting reversible contraceptives (LARCs) [18–20], they have not quantified women’s preferences or assessed their willingness to pay (WTP) using discrete choice experiment (DCE) methods. This study therefore addresses a critical evidence gap by applying DCE to evaluate Ethiopian women’s preferences for LARCs, including key attributes such as method effectiveness, side effects, provider type, method duration, and service cost.

In summary, this study aims to assess reproductive-age women’s preferences for LARC methods in Ethiopia using a Discrete Choice Experiment. The results will quantify attribute-level trade-offs and WTP, offering evidence to guide policymakers, health service planners, NGOs, and researchers in designing demand-driven interventions to enhance LARC utilization and reduce unmet contraceptive needs.

## Methods and materials

### Study setting and period

The study was conducted in Gondar town, located in the Central Gondar zone of the Amhara regional state, approximately 750 km northwest of Addis Ababa. Gondar town has a total population of 333,103, including approximately 78,546 women of reproductive age, distributed across six sub-cities and 22 Kebele. The town is equipped with eight public health centers, one comprehensive specialized hospital, and one general hospital. From March 28, 2023, to April 28, 2023, the research was carried out in five selected public health facilities: Maraki Health Center, Gondar Health Center, Azezo Health Center, Teda Health Center, and Gondar University Comprehensive Specialized Referral Hospital, situated in Gondar, Northwest Ethiopia.

### Study design

An institution-based cross-sectional study design utilizing the discrete choice experiment (DCE) approach was employed to assess women’s preference for LARCs in public health service facilities in Gondar town. DCE is a quantitative method that uncovers personal preferences by presenting participants with various hypothetical alternatives, enabling researchers to gauge how individuals value specific program features, goods, or services [22, 23]. It aids in understanding priority setting in health service provision by revealing people’s preferences for different treatment options and the trade-offs they are willing to make [25]. With its ability to anticipate real-world judgments, DCE has been widely applied in medicine to elicit stated preferences for health and healthcare [32, 33]. Participants are tasked with selecting between fictitious events with varying characteristics, showcasing the relative importance of each attribute and the consideration given to trade-offs. As healthcare professionals increasingly seek to maximize the impact of health-related activities, DCEs are becoming more prevalent in healthcare and public health contexts [34].

### Identification of attributes and levels

The discrete choice experiment (DCE) methodology involves a systematic six-step process to develop a locally relevant and well-designed study. These steps include formative work for attribute identification, attribute selection (with a limit of fewer than ten attributes), attribute level selection with realistic ranges and 2–4 levels per attribute, selection of DEC design (fractional vs. full factorial), determination of attribute level combinations, and enhancement and assessment of tool comprehensibility through planning tool appearance and pretesting [34, 35]. In the context of this study on long-acting reversible contraceptive (LARC) family planning methods, nine significant attributes were identified from various literature sources [28, 29, 36–39], and expert opinions were sought to refine them. Experts from departments such as Reproductive Health, Health Economics, and Midwifery were involved in evaluating and ranking these attributes, resulting in the selection of the top six attributes: effectiveness [4, 27, 28, 37, 40], effect on bleeding [27, 28, 31, 36, 37, 39], effect on weight [28, 31, 37], duration [37], cost [41], and service provider type [29, 42, 43]. Attributes ranked lower by experts were excluded to reduce cognitive burden, as recommended in DCE design guidelines. Practical and reliable levels were assigned to each attribute, ranging from two to four, and these attributes and levels were meticulously detailed in Table 1 for reference.

**Table 1:** Attributes and levels for hypothetical LARC family planning methods at Gondar town selected public health service facilities 2023

Attribute	Level	Definition of levels
Effectiveness	Effective	1–2 pregnancies per 100 women per year
	Very effective	≤ 1 pregnancy per 100 women per year
Effect on weight	No effect	LARC has no change in body weight
	Slight weight increase	Slight weight increase of 3 to 6 kg per year
Effect on bleeding Pattern	No bleeding	High chance of amenorrhea (no periods) in the long term
	Irregular bleeding	Light, irregular menstrual bleeding that occurs unpredictably in timing and flow
	Heavy bleeding	Menstrual periods that are heavier in flow and last longer than normal
Service Provider type	Health Extension Worker/HEW	Provides basic curative and preventive services (family planning, vaccination, health education)
	Health officer/HO	Undergo 4-year training to provide clinical & public health services in rural hospitals & HC.
	Midwife Nurse	Provides maternal and newborn care during pregnancy, delivery, and postpartum.
	Medical Dr.	Dr. is Licensed physician trained to provide full medical care.
Duration	Relatively short	Prevents pregnancy for 3–5 years
	Relatively long	Prevents pregnancy for 5–12 years
Cost of LARC	Free provision	No out-of-pocket cost (covers contraceptive, service, and card fees)
	100 ETB	Out-of-pocket cost of 100 ETB
	500 ETB	Out-of-pocket cost of 500 ETB

ETB = Ethiopian Birr. 1$ could be exchanged for about 54 Ethiopian Birr at the time of the study

### Experimental design of choice set

The study employed a strategic experimental design to efficiently manage the numerous combinations of attributes and levels involved in assessing preferences for long-acting reversible contraceptive (LARC) methods. Instead of rating every potential combination in a full factorial design, SAS software generated a fractional factorial design, reducing the combinations to a manageable level [44, 45]. With six attributes and varying levels totaling 288 possible combinations, the fractional factorial design produced 24 comparisons with two forced hypothetical LARC alternatives. A forced-choice format was deliberately selected, requiring respondents to choose between the two alternatives without an opt-out. This approach was chosen to maximize statistical efficiency, avoid excessive non-choice responses that could limit estimation of preferences, and focus specifically on understanding women’s trade-offs between LARC attributes rather than the broader decision of whether to use contraception. To reduce respondent burden, the experimental design (24 choice tasks, each with two hypothetical LARC alternatives) was divided into two blocks. Respondents were randomly assigned to one block and therefore completed 12 Choice sets each (equivalent to 24 profiles per respondent). Of the 344 respondents, 172 were assigned to Block 1 and 172 to Block 2 through simple randomization. In total, the design comprised 48 unique profiles and generated 8,256 profile evaluations across the sample (344 respondents’ × 12 tasks × 2 alternatives). These comparisons were organized into 48 scenarios (choice cards) within the questionnaire.

The properties of the well-designed questionnaire were assured using the criteria of orthogonality, level balance, and minimizing overlap. Orthogonality ensures that attributes vary independently, allowing the effect of each attribute on choice to be estimated without confounding. Level balance ensures that all levels of each attribute occur approximately equally often across choice tasks, giving each level an equal chance of being chosen. Minimizing overlap ensures that alternatives in a choice set do not have identical levels for a given attribute, which maximizes the trade-off information obtained from respondents. These design features improve data quality and enhance respondents’ comprehension and decision-making. Table 2 displays an exemplary choice scenario crafted for this study.

**Table 2:**
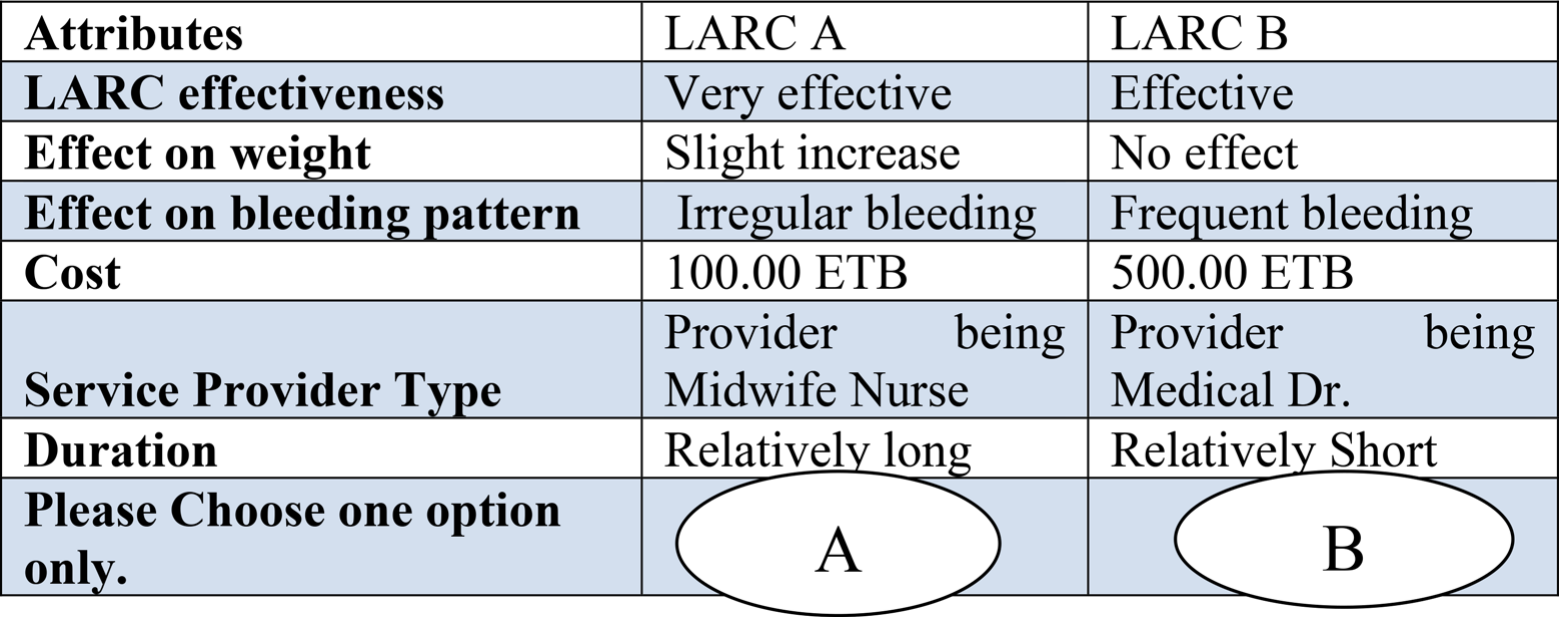
Sample choice set scenarios

## Population

### Source population

The study’s source population was all reproductive age women (15–49 year) who attended for the Contraceptive Service in Gondar town public health facilities. 

## Study population

The Study population was all reproductive-age women who attended for contraceptive services during the study period at selected public health service facilities In Gondar, North West Ethiopia, 2023.

## Inclusion and exclusion criteria

### Inclusion criteria

Reproductive age women who came for contraceptive services at the selected public health facilities during the data collection period were included in the study.

### Exclusion criteria

Women who were critically ill, unable to communicate, refused to participate, or were temporary residents of Gondar town at the time of data collection were excluded from the study.

## Sample size determination and sampling procedures

### Sample size determination

The sample size for the Discrete Choice Experiment (DCE) was calculated using Orme’s rule of thumb (nta/c ≥ 500), which considers the number of participants, choice tasks, alternatives, and attribute levels [46–48]. While not entirely precise, it provided an initial estimation.

A more common parametric approach was used for a more accurate calculation: [49]

$$N\geq\left(\frac{z^2q}{rp\alpha^2}\right)$$

A preference’s choice share, or p, is the proportion of a single attribute to the total attribute; in this instance, p is 1/6. In this case, there are six attributes. Assume that the population is heterogeneous (*p* = 0.167, q = 0.833). With 95% confidence, Z = 1.96, and the allowable error (a) is 5%.

The design included 24 replications (r) and 24 choice sets created using a fractional factorial design. The calculated sample size was 319. Taking into consideration a 10% non-response rate, equivalent to 32 participants, it was calculated that a total of 351 respondents were needed for the study. In the absence of prior preference estimates, a proportion-based formula was used as a practical approach. A total of 344 participants (98% of the target) were enrolled, sufficient for reliable preference estimation based on typical DCE sample sizes of 200–400 participants.

### Sampling procedure

Gondar town has ten public health facilities offering family planning services, including the University of Gondar Specialized Hospital, Ayra General Hospital, and several health centers. For this study, five facilities were selected using the World Health Organization’s guidelines to assess half of the available institutions.

The facilities chosen through a lottery method were the University of Gondar Hospital, Maraki Health Center, Azezo Health Center, Gondar Health Center, and Teda Health Center. The size of the sample was determined by allocating it proportionally according to the number of family planning users that each facility has been receiving daily in recent times.

**Fig. 1:**
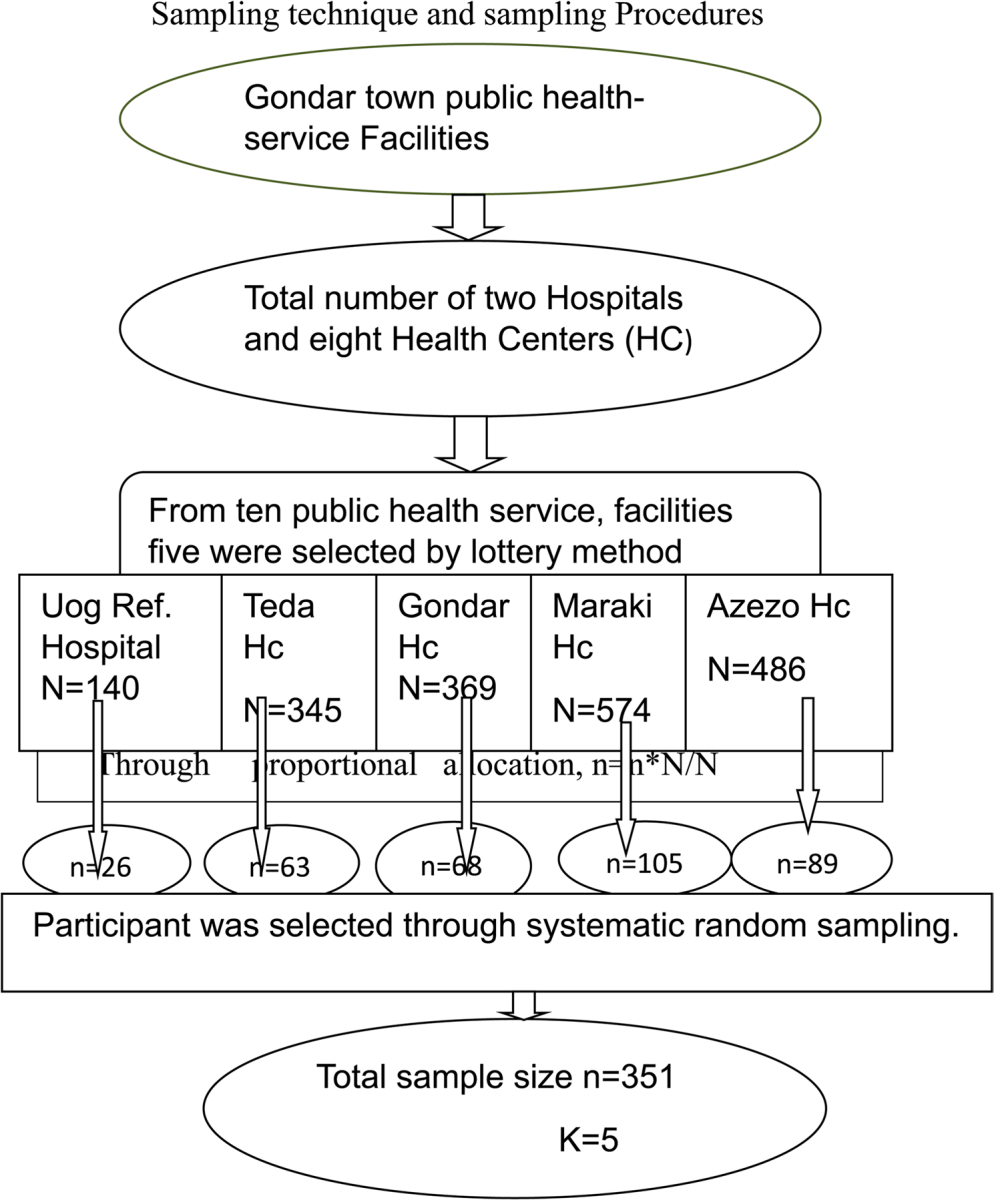
Illustrates the schematic diagram of the sampling process conducted in public health service facilities within Gondar town in the year 2023

Participants were selected using systematic random sampling. Every fifth woman receiving contraceptive services was chosen, starting with the first participant selected through a lottery method until the desired sample size was reached for each facility. The sampling procedure, including the selection of health facilities and participants, is outlined in Fig. 1.

### Data collection procedures

Data was collected by five trained midwife nurses using the Kobo Toolbox app on smartphones. Two supervisors specializing in Public Health oversaw the process to ensure data accuracy. Socio-demographic information was collected using a tool adapted from a Nigerian study [50]. A choice set, developed with SAS JMP software, focused on selected LARC preferences. The questionnaire combined socio-demographic data with main choice questions on LARC attributes. Initially prepared in English, the questionnaire was translated into Amharic and returned to English to check consistency. The DCE questionnaire included a warm-up and main choice scenario, which were presented to familiarize participants. Respondents chose between two hypothetical LARCs characterized by different attributes, as shown in Table 2.

### Data quality assurance and management

Data quality was ensured through extensive training for data collectors and supervisors on study objectives, techniques, tools, respondent interaction, data confidentiality, and participant rights. A pretest of 10% (35 participants) was conducted one week prior to data collection at Debre Tabor Health Center. Pretest participants resembled the study group, allowing for modifications and consistency checks, such as computing Cronbach’s α (7.12).

The structured questionnaire included socio-demographic data adapted from a Nigerian study, with adjustments for the Ethiopian context. Main choice sets were developed using SAS JMP software.

Surveys and questionnaires were initially crafted in English before being translated into Amharic and then back into English to ensure uniformity and accuracy. Since DCEs involve hypothetical choices, internal and external validity checks were vital for data quality assurance. Face-to-face interviews yielded a 98% response rate, with 344 respondents providing 8256 observations across 24 choice tasks. Respondents were re interviewed to verify their answers for consistency.

Data collected via the Kobo toolbox was coded and entered into Microsoft Excel, where it was cleaned and checked for missing values.

### Statistical analysis

A mixed logit model (MXL) was used to assess reproductive-age women’s preferences for LARC methods. This approach accounts for parameter variability across a population and correlated responses from individuals [51, 52]. Most attributes were coded as dummy variables, while the cost of LARC was treated as a continuous, fixed variable. A choice model was set up, and the main effects were analyzed using mixed logit, with a significance threshold of *p* < 0.05. The goodness of fit was evaluated through log-likelihood and pseudo-R-squared values.

Multicollinearity among the independent attribute variables was assessed using Variance Inflation Factors (VIFs), which are recommended as a more robust diagnostic than simple correlation matrices [53]. All VIF values were below 2 (mean VIF = 1.29), indicating no evidence of problematic multicollinearity. The willingness-to-pay (WTP) for level *m* of attribute *n* was calculated as the part-worth utility of that attribute level divided by the price coefficient: WTPnm = βnm/Βp [54].

SAS software was used for the experimental design and preparation of choice sets due to its robust procedures for orthogonal and balanced fractional factorial designs. Mixed logit (MXL) models were estimated in Stata 17, which provides well-documented routines (mixlogit) for random-parameter models and panel data. This combination leveraged the strengths of each software package, ensuring efficient design and robust preference estimation [55, 56].

## Results

### Socio-demographic characteristics of the study participant

A total of 344 participants, with a 98% response rate, were analyzed. The mean age of the respondents was 27.52 ± 5.70 years. Most participants, 335 (97.38%), lived in urban areas, and 75.8% were married. In terms of education, 57 respondents (16.57%) were illiterate, and 136 participants (39.53%) were housewives by occupation (refer to Table 3).

**Table 3:** Socio-demographic characteristics of the participants on Long-acting reversible contraceptive preference in Gondar town selected public health facilities Northwest Ethiopia, 2023 (N=444

Variables	Category	Frequency	Percent (%)
Age in years	15–24	107	31.1
	25–34	179	52
	35–49	58	16.9
Residency	Urban	335	97.38
	Rural	9	2.62
Educational status	Cannot read & write	57	16.6
	Can read & write	12	3.5
	Primary school	56	22.6
	Secondary school	95	27.6
	Collage & above	104	30.2
Marital Status	Not married	70	20.3
	Married	261	75.9
	Divorced	11	3.2
	Widowed	2	0.6
Occupation	Government employee	42	12.20
	Privet employee	25	7.28
	Non-government employee	4.07	14
	Merchant	42	12.21
	Farmer	3	0.87
	Homemaker/Housewife	136	39.53
	Student	43	12.50
	Daily Laborer	31	9.01
	Other	8	2.33
Number of children	No(0)	100	29.1
	1–2	155	45
	3–5	87	25
	> 5	2	0.58
Income	< 1000	51	14.8
	1001–2000	67	19.5
	> 2001–3000	75	21.8
	> 3000	151	43.9

### Contraceptive utilization information

Most respondents, 212 (61.6%), currently use injectable Depo contraceptives. Additionally, 20% use oral contraceptives (OC), 16% use implants, and 2% use intrauterine devices (IUCD). A majority, 194 (56.4%), chose their current contraceptive due to minimal side effects, while 115 (33.43%) based their choice on duration. Others opted for their method due to easy manageability (4.65%) or effectiveness (3.49%).

### Main-effect model

Table 4 summarizes the mixed logit model’s coefficients, standard errors (SE), and willingness-to-pay (WTP) estimates for attributes influencing women’s preferences for long-acting reversible contraceptives (LARC) methods.

**Table 4:** Mixed logit model reflecting women’s Stated preferences for LARCs in selected health service facilities in Gondar town Northwest Ethiopia 2023 (N=344)

CHOICE	Coefficient Std. Errs. Z P>|z| [95% CI]
Mean	
Cost	−0.0017 0.00012 −13.35 0.000 −0.0019 −0.0014
Very Effective	0.1329 0.0415 3.20 0.001 0.0516 0.2142
Slight weight increase	0.2564 0.0436 5.88 0.000 0.1709 0.3418
No menstruation bleed	0.1233 0.0492 2.51 0.012 0.0269 0.2198
Heavy bleed	−1.1396 0.0925 −12.32 0.000 −1.3209 −0.9583
HO service provider	0.3557 0.0742 4.79 0.000 0.2102 0.5011
Midwife service provider	0.8747 0.0834 10.48 0.000 0.7111 1.0382
Doctor service provider	0.4717 0.0711 6.64 0.000 0.3325 0.6109
5 to 10-year prevention	−0.2305 0.0506 −4.55 0.000 −0.3297 −0.1313
SD	
Very Effective	0.1841 0.1172 1.57 0.116 −0.0456 0.4139
Slight weight gain	−0.0986 0.2309 −0.43 0.669 −0.5512 0.3541
No menstruation bleed	−0.2410 0.1279 −1.88 0.059 −0.4917 0.0096
Heavy bleed	0.8566 0.1069 8.01 0.000 0.6471 1.0661
HO service provider	0.1494 0.2393 0.62 0.532 −0.3198 0.6186
Midwife service provider	0.2761 0.1686 1.64 0.102 −0.0543 0.6064
Doctor service provider	0.2763 0.1398 1.98 0.048 0.0023 0.5503
5 to 10-year prevention	0.5447 0.0641 8.49 0.000 0.4189 0.6703

Note: Number of respondents =344; Number of observation =8256; log likelihood = −2466.1919; LR chi2 (8) =82.08; probability *>χ *2= 0.0000.

Abréviations: *SD *Standard Déviation;, *CI* Confidence interval;, *Std* *Err* Standard Error, *LR*. Likelihood ratio

Cost was a major factor. As method price increased, selection probability declined significantly (β = −0.0017; *p* < 0.001). Women’s WTP estimates indicated high price sensitivity. Specifically, women were willing to pay approximately 528 ETB [10.13 USD] more to receive LARC services from a midwife rather than a health extension worker. In the Ethiopian context, this represents a considerable financial burden, potentially equivalent to several days of income for low-income women. Preferences for doctor-provided services reflected a WTP of approximately 284 ETB [5.45 USD], and for health officer-provided services, approximately 214 ETB [4.11 USD]. These findings highlight the financial barriers to LARC access without external subsidies.

Method effectiveness also influenced preferences. Women favored highly effective methods, defined as those preventing over 99% of pregnancies (β = 0.1329; *p* = 0.001). However, variation in preference for effectiveness was minimal across respondents.

Side-effect profiles were significant. Women preferred methods associated with slight weight gain (defined as less than 2 kg over six months) (β = 0.2564; *p* < 0.001) and those causing no menstrual bleeding (amenorrhea during contraceptive use) (β = 0.1233; *p* = 0.012). In contrast, methods linked to heavy and prolonged bleeding were strongly avoided (β = −1.1396; *p* < 0.001). Notably, preferences regarding bleeding-related side effects varied across subgroups.

Service provider type was a key determinant. Midwife-provided services were most preferred (β = 0.8747; *p* < 0.001), with women willing to pay significantly more for midwife-led provision, as noted above. Preferences for doctor-provided (β = 0.4717; *p* < 0.001) and health officer-provided services (β = 0.3557; *p* < 0.001) were also significant, though with lower WTP values.

Duration of contraceptive protection affected choices. Women preferred short-term methods and actively avoided those lasting 5 to 10 years (β = −0.2305; *p* < 0.001). Considerable preference heterogeneity was observed for this attribute, indicating divergent attitudes toward long-term contraceptive commitment.

### The relative impact of the attributes on preference

Table 5 summarizes the relative importance of attributes influencing women’s preferences for long-acting reversible contraceptives (LARCs) in selected health facilities of Gondar Town, Northwest Ethiopia (*N* = 344). Heavy menstrual bleeding emerged as the most influential attribute, contributing 34.4% to the explained variation, indicating that avoiding this side effect is a primary concern in LARC selection. Cost followed as the second most important factor, accounting for 25.3% of the relative effect, highlighting the role of affordability in decision-making. The preference for receiving services from a midwife nurse ranked third (15.5%), followed by the preference for long-duration contraceptive methods (7.9%) and service provision by a medical doctor (7.1%). Less influential attributes included slight weight gain (4.4%), service provision by a health officer (3.0%), very high contraceptive effectiveness (1.4%), and absence of menstruation bleeding (1.0%). These results suggest that women’s choices are primarily driven by concerns over side effects, cost, and provider type.

**Table 5:** Ranking of attributes level importance for women’s LARC preference in selected health service facilities in Gondar town Northwest Ethiopia 2023 (N=344)

Attribute level Omitted from Analysis	Log-likelihood	Partial Effect Change in log-likelihood	Relative effect% sum of the change in log-likelihood	Cumulative (%)	Order of impact
None	−2466.1919	-	-	-	-
Heavy bleeding	−2611.5325	−145.3406	0.344	0.344	1
Cost	−2573.1156	−106.9237	0.253	0.597	2
Midwife	−2531.5174	−65.3255	0.155	0.752	3
Dun 5 to 12 year	−2499.7717	−33.5798	0.079	0.831	4
Medical Dr.	−2492.1462	−29.9543	0.071	0.902	5
Slight wt. gain	−2484.7608	−18.5689	0.044	0.946	6
Health officer	−2478.7291	−12.5372	0.03	0.976	7
Very Effective	−2472.2097	−6.0178	0.014	0.99	8
No bleeding	−2470.3498	−4.1579	0.01	1.00	9

### Probability of LARC take-up

As presented in Table 6, higher costs significantly reduced the probability of LARC uptake, with decreases of 8.0% at a price of 100 ETB and 39.2% at 500 ETB. Similarly, heavy menstrual bleeding and a 5–10-year pregnancy prevention method reduced uptake probabilities by 51.5% and 11.5%, respectively. In contrast, several factors were associated with increased uptake. A very effective LARC increased uptake probability by 6.6%, slight weight increase by 12.8%, and absence of menstruation bleeding by 6.2%. Service provider type also influenced uptake, with provision by health officers increasing uptake by 17.6%, midwife nurses by 41.1%, and medical doctors by 23.2%.

**Table 6:** Probability of LARC taken by women in selected health service facilities in Gondar town Northwest Ethiopia 2023 (*N* = 344)

Probability of LARC uptake compared to baseline					
LARC attribute Levels	Coefficient	Std. errs.	Z	P>|z|	[95% conf. interval]
Cost = 100 ETB	−0.0826	0.0062	−13.41	0.000	−0.0947 −0.0706
Cost = 500 ETB	−0.3920	0.0263	−14.93	0.000	−0.4435 −0.3405
Very effective	0.0664	0.0207	3.21	0.001	0.0259 0.1068
Slight weight increase	0.1275	0.0214	5.95	0.000	0.0855 0.1695
No bleeding	0.0616	0.0245	2.51	0.012	0.0135 0.1096
Heavy bleeding	−0.5152	0.0339	−15.17	0.000	−0.5818 −0.4486
Provider HO	0.1760	0.0359	4.89	0.000	0.1055 0.2465
Provider Midwife Nurse	0.4115	0.0346	11.87	0.000	0.3435 0.4793
Medical Dr.	0.2316	0.0336	6.89	0.000	0.1657 0.2975
Duration Relatively long	−0.1147	0.0249	−4.59	0.000	−0.1637 −0.0658

## Willingness to pay (WTP) for LARC attributes

Table 7 presents the estimated willingness to pay (WTP) values for various long-acting reversible contraceptive (LARC) attributes among reproductive-age women. The results indicate that women are willing to pay an additional 80.21 ETB (95% CI: 30.78, 129.65) for highly effective LARC methods, 154.77 ETB (95% CI: 99.48, 210.06) to avoid slight weight gain, and 74.43 ETB (95% CI: 15.52, 133.35) for LARC methods that result in no menstruation bleeding. Preferences related to service providers showed that women were willing to pay 527.98 ETB (95% CI: 415.92, 640.04) for services provided by midwife nurses, 284.75 ETB (95% CI: 196.68, 372.82) for medical doctor services, and 214.70 ETB (95% CI: 123.23, 306.16) for health officer services. In contrast, attributes such as heavy menstrual bleeding and long-duration pregnancy prevention (3 to 5 years) were associated with negative WTP values of −687.89 ETB (95% CI: −831.44, −544.33) and − 139.13 ETB (95% CI: −200.23, −78.03), respectively, indicating strong disutility and a preference to avoid these options.

**Table 7:** Women’s WTP for LARC women in selected health service facilities in Gondar town Northwest Ethiopia 2023 (*N* = 344)

Variable	Coefficient	Std. errs.	Z	*P*>|z|	[95% conf. interval]
Contraceptive Effectiveness Very effective	80.21	25.2219	3.18	0.001	30.77914 129.6472
Effect on weight Slight weight gain	154.77	28.2111	5.49	0.000	99.47652 210.062
Effect on bleeding					
NO Bleeding	74.43	30.0601	2.48	0.013	15.5146 133.3481
Heavy bleeding	−687.89	73.2429	−9.39	0.000	−831.44 −544.3339
Service provider					
Health officer	214.70	46.6682	4.60	0.000	123.228 306.1638
Midwife Nurse	527.98	57.1735	9.23	0.000	415.9234 640.0394
Medical Dr.	284.75	44.9358	6.34	0.000	196.68 372.8252
Duration					
Relatively long Year	− 139.13	31.1737	−4.46	0.000	−200.23 78.03

### Subgroup analysis

Table 8 presents the subgroup analysis of reproductive-age women’s choice of long-acting reversible contraception (LARC) based on their marital status

**Table 8:** Subgroup analysis

CHOICE	Coefficient	Std. Errs.	Z	P>|z|	[95% CI]
Mean					
Married_dummy	−18.9469	5743.5	−0.00	0.997	−11276 11238.11
Divorced_dummy	−19.1133	5743.5	−0.00	0.997	−11276.17 11237.94
Very effective	0.1156	0.04	3.14	0.002	0.0434 0.1879
Slight weight gain	0.1786	0.04	4.89	0.000	0.1071 0.2502
No bleed	0.1263	0.04	2.94	0.003	0.0421 0.2105
Heavy bleed	−0.9270	0.06	−15.91	0.000	−1.0413 −0.8128
Service provider HO	0.3059	0.07	4.60	0.000	0.1757 0.4361
Service provider _Midwife	0.7745	0.07	10.74	0.000	0.6331 0.9158
Service provider _Dr.	0.3839	0.06	6.19	0.000	0.2624 0.5054
5 to 12 year duration preventive	−0.1746	0.04	−4.85	0.000	−0.2451 −0.1039
Cost	−0.0014	0.00009	−14.35	0.000	−0.0016 −0.0012
SD					
Cost	−0.00003	0.0008	−0.04	0.969	−0.0015 0.0015

The subgroup analysis reveals that unmarried women are more likely to choose long-acting reversible contraception (LARC) compared to both married and divorced women. Specifically, married women are less likely to opt for LARC than their unmarried counterparts, and divorced women also show a lower likelihood of choosing LARC compared to unmarried women.

## Discussion

Heavy menstrual bleeding emerged as the most significant barrier to long-acting reversible contraceptive (LARC) uptake in this study, reducing the likelihood of adoption by over 50% compared to methods causing irregular bleeding. This finding reflects a strong demand for side-effect management, consistent with previous studies from Ethiopia [36] and Europe [57], where bleeding concerns were key contributors to early method discontinuation. This also aligns with findings from several prior studies conducted both in Ethiopia and other low- and middle-income countries. For instance [58] and [59] reported that excessive bleeding and extended menstruation, were among the most frequently cited reasons for early Implanon removal. The substantial willingness to pay approximately 688 ETB (13.24 USD) to avoid heavy bleeding further illustrates the disutility placed on this side effect. Interventions that incorporate structured counseling, visual decision aids, and peer education could help mitigate bleeding-related fears and improve uptake [37, 60]. Addressing misconceptions and providing clear information about bleeding patterns may reduce discontinuation risks. From a policy and economic perspective, prioritizing bleeding management aligns health system efficiency with women’s expressed preferences, potentially lowering costs related to unintended pregnancies, repeat visits, and method replacement, making it a cost-effective component of LARC programs [61, 62].

Heavy menstrual bleeding, cost, and provider type (midwife/nurse) were the most influential factors shaping women’s preferences for long-acting reversible contraceptives (LARCs). While amenorrhea (no menstrual bleeding) ranked lowest in preference impact—with a 12.33% preference share and a 6.64% probability of uptake—it still influenced women’s willingness to pay 74.43 ETB to avoid irregular bleeding, illustrating the economic trade-offs women consider in contraceptive choice. These findings suggest that menstrual changes are less decisive than side effects, affordability, and trusted providers in initial method selection, yet remain important for satisfaction and continuation. From a health economics perspective, the results indicate that interventions targeting provider training, cost reduction, and counseling on side effects could increase LARC uptake and continuation. This aligns with evidence from Kenya, Uganda, and Ethiopia, where bleeding patterns affect continuation but rarely dominate initial method choice [63–65].

Service provider type was another key determinant of women’s LARC preferences. The role of service provider type in shaping LARC preferences has direct implications for health workforce planning and resource allocation. In this study, receiving services from midwife nurses increased LARC uptake probability by over 40%, while medical doctors were associated with an uptake increase of approximately 23%, compared to health extension workers. These preferences are consistent with findings from Kenya [66], England [67], and Australia [28], where higher provider qualifications were associated with increased trust and service acceptance. Given Ethiopia’s reliance on health extension workers for contraceptive service delivery, these findings suggest that expanding midwife-led services and enhancing health extension workers’ training could improve service credibility and build client trust. Investing in professional provider capacity may therefore strengthen the family planning service delivery model.

Provision by health officers significantly increased uptake (by 17.6%), and women expressed a willingness to pay about 215 ETB (3.9 USD) for their services. While midwives remained the most preferred providers, this finding indicates that women also place considerable trust in health officers, underscoring their potential role in expanding contraceptive access when midwives are less available. Consistent with this, women emphasized the central role of health providers in shaping their family planning decisions; however, provider bias against preferred methods was reported to hinder both uptake and satisfaction [68].

Cost was another significant determinant of women’s contraceptive choices. This study highlights significant price sensitivity in LARC demand. Even a modest user fee of 100 ETB (1.92 USD) led to an 8.3% reduction in uptake, with higher fees producing sharper declines. An increase of 500 ETB in service cost resulted in an almost 40% reduction in the likelihood of LARC utilization. Women’s willingness to pay more for trusted providers 528 ETB (10.15USD) for midwife services and 285 ETB (5.48 USD) for medical doctors but less for the methods themselves suggests that women prioritize service quality over product characteristics. This behavior indicates high price elasticity of demand for LARCs, consistent with studies from Nigeria [31], Sub-Saharan Africa [69], and the United States [70], where direct costs act as barriers to contraceptive use. Maintaining free LARC provision should therefore be a key policy objective. However, if full subsidization is financially unsustainable, alternative financing strategies such as targeted voucher programs or integration of contraceptive services into Ethiopia’s community-based health insurance scheme could help protect economically vulnerable LARC utilizers, such strategies have proven feasible in similar low-resource settings. High price sensitivity indicates that such policies could prevent reductions in uptake while maintaining program cost-effectiveness.

Beyond cost and provider factors, side-effect profiles and method effectiveness also influenced preferences. Women preferred LARCs associated with modest weight gain (3–6 kg per year), with a 25.6% higher preference relative to weight-neutral methods, and expressed a WTP of 154.77 ETB (2.7 USD) for such methods. This contrasts with findings from Australia and Nigeria [28, 31], where weight-related side effects discouraged LARC use differences that may reflect cultural norms and perceptions of body image within different populations. From a policy perspective, these findings suggest that pricing strategies or counseling interventions could be tailored to accommodate cultural attitudes toward body changes associated with contraceptive use. Method effectiveness also positively influenced preferences, increasing uptake probability by 6.6% and generating a WTP of approximately 80.21 ETB (1.4 USD) for high-efficacy methods. These findings align with prior evidence from England and Australia [20, 21], where reliability is a priority attribute. The economic valuation of these attributes suggests that subsidy design should account not only for method type but also for user-valued features such as effectiveness and side-effect profiles.

Duration of protection influenced women’s preferences, with 3–5 year methods favored over longer-term (5–10 year) options. Longer-duration LARCs reduced uptake likelihood by 11.47%, and women were willing to pay 139 ETB (2.4 USD) to avoid extended protection. This preference may reflect concerns about long-term fertility and the desire for flexibility. Similarly, a previous study found that only 8.4% of short-acting contraceptive users intended to adopt IUCDs, mainly due to unfavorable attitudes and negative social norms [71]. Both findings suggest that misconceptions and social influences contribute to resistance toward long-acting methods, highlighting the need for targeted counseling on method duration.

Subgroup analysis revealed that unmarried women were more likely to prefer LARCs compared with married or divorced women. This pattern likely reflects differences in reproductive intentions, autonomy, and social expectations across marital status. In contrast, a study in Malawi reported that unmarried women had lower LARC use compared to married women, likely due to higher sexual activity and greater acceptance of LARCs among married women [61]. These findings highlight the need for family planning services that are tailored to marital-status-specific needs, providing youth- and marital-status-sensitive counseling to enhance responsiveness and uptake among diverse groups of women.

Overall, these findings confirm that women’s contraceptive preferences are shaped by a combination of cost, provider type, side-effect profiles, effectiveness, duration, and Socio-demographic factors. By explicitly linking willingness-to-pay values to potential policy actions such as prioritizing midwife-led delivery, financing bleeding management interventions, and designing targeted subsidies this study provides evidence that can inform demand-responsive, efficient, and equitable resource allocation in Ethiopia’s reproductive health services.

### Limitations of the study

This study has some limitations. Subgroup analyses between urban and rural participants, as well as between hospital and health center settings, were not feasible due to the small sample size of rural residents and hospital-based clients (fewer than 30 in each subgroup). Future studies should explore these variations to identify potential preference heterogeneity. The study was also limited to public health facilities in Gondar town, excluding women using private sector services. Inclusion of both public and private sector participants in future research would improve generalizability. Additionally, the lack of comparable studies using stated or revealed preference methods within the Ethiopian contraceptive service context limits direct comparisons with other settings.

Despite these constraints, this study provides important demand-side insights into women’s preferences for LARCs. By quantifying how specific attributes and service factors influence choice and willingness to pay, the findings contribute valuable evidence to guide contraceptive policy and financing strategies in resource-constrained settings.

### Conclusion and policy recommendations

This study demonstrates that women’s preferences for long-acting reversible contraceptives (LARCs) in Ethiopia are shaped by side-effect profiles, provider type, method effectiveness, duration of protection, cost sensitivity, and socio-demographic characteristics, each reflected in willingness-to-pay estimates. Heavy menstrual bleeding emerged as the strongest barrier to uptake, whereas midwife-led services, high-efficacy methods, and medium-term (3–5 year) protection were consistently preferred. Women placed greater value on provider quality than on product characteristics, and the observed price sensitivity highlights the potential for financial barriers to reduce uptake and continuation. Subgroup analysis further showed that marital status influences preferences, with unmarried women more likely to favor LARCs, underscoring the importance of tailored counseling and service delivery.

These findings indicate that prioritizing bleeding management through counseling, treatment, and provision of predictable-bleeding or bleed-free methods could improve continuation and reduce unintended pregnancies. Expanding midwife-led services, recognizing the role of health officers, and strengthening health extension worker training offer cost-efficient task-shifting strategies that build client trust. Maintaining free provision, or implementing targeted subsidies and community-based health insurance schemes, is essential to protect low-income women. Incorporating women’s stated preferences into financing, workforce planning, and service design can enhance efficiency, equity, and responsiveness in Ethiopia’s reproductive health programs, with bleeding management and midwife-led provision emerging as the most cost-effective levers.

## Supplementary Information

Supplementary Material 1

## Data Availability

The information backing up the results of this research can be provided upon request from the author responsible for correspondence.

## Abbreviations

DCE: Discrete choice experiment
ETB: Ethiopian birr
HC: Health center
HO: Health officer
HEW: Health extension worker
IUCD: Intrauterine contraceptive device
LARC: Long acting reversible contraceptive
MMR: Maternal mortality rate
MOH: Ministry of health
MXL: Mixed logit model
WHO: World health organization
WTP: Willingness to pay
